# Dissociation of modular total hip arthroplasty at the neck–stem interface without dislocation

**DOI:** 10.1007/s10195-011-0172-9

**Published:** 2011-12-08

**Authors:** A. Kouzelis, C. S. Georgiou, P. Megas

**Affiliations:** Department of Orthopaedics and Traumatology, University Hospital of Patras, 26504 Rio, Patras, Greece

**Keywords:** Total hip arthroplasty, Femoral neck–stem dissociation, Surgical revision

## Abstract

Modular femoral and acetabular components are now widely used, but only a few complications related to the modularity itself have been reported. We describe a case of dissociation of the modular total hip arthroplasty (THA) at the femoral neck–stem interface during walking. The possible causes of this dissociation are discussed. Successful treatment was provided with surgical revision and replacement of the modular neck components. Surgeons who use modular components in hip arthroplasties should be aware of possible early complications in which the modularity of the prostheses is the major factor of failure.

## Introduction

Modular femoral components have the advantages of reducing the need to stock many stem and head sizes and of allowing the final choice of neck length and head size to be made after stem implantation. Neck orientation can also be changed after implantation, which as is well known can be the cause of early dislocation. The incidence of postoperative dislocation of modular total hip arthroplasty (THA) varies from 0.5% to 4% [1]. Dissociation at the neck–stem interface is rare. To the best of our knowledge, only three case reports have been published [2–4], but they pertained to dissociation at the neck–head interface. We report a case of dissociation at the neck–stem interface without hip dislocation that occurred during walking, and we discuss the causes of dissociation as well as strategies to avoid and treat this complication.

## Case report

**Fig. 1:**
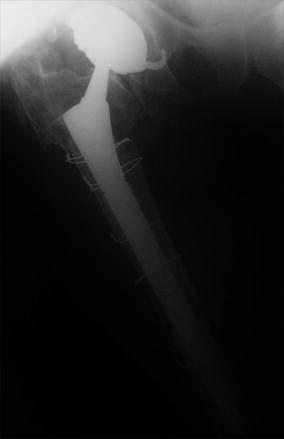
Radiograph after the first operation reveals good relationship of the total hip prosthesis with acetabular and femoral bones

A 72-year-old man was received a right THA in 1996. THA revision was performed in our institution in 2005 due to aseptic loosening of both components. Intraoperatively, extraction of the acetabular shell revealed serious bone loss, so we decided to use a jumbo acetabular component (Procotyle, Wright). Allograft augmentation of the acetabulum was also used to repair the acetabular bone defect. The acetabular shell was 60 × 68 mm in outer diameter, and additional fixation was achieved with three cancellous screws. The polyethylene liner was group 2, 15°, 28 mm in inner diameter. For the femoral component, which was fully porous coated and therefore distally fixed, we used a modular stem (Profemur-R, Wright). The open-book technique was used to extract it, and a transverse osteotomy just under the tip was also made, which we use in such cases to avoid distal extension of the osteotomy (open-book technique) and to preserve good bone stock for the distally fixed stem. Postoperative radiographic control revealed adequate positioning of the THA components (Fig. 1). The usual protocol for THA postoperative treatment was used, and patient mobilization began on the second postoperative day. The patient was discharged on the eighth postoperative day, fully mobilized (partial weight bearing) and without residual problems. The patient gave informed consent to publish this case.

**Fig. 2:**
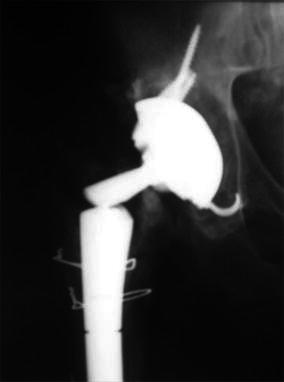
Radiograph shows dissociation of the femoral neck–stem interface

The usual clinical and radiographic follow-up during the first and third months (Fig. 4b) was normal. The patient was satisfied with the result of the operation and was mobilized with two canes (according to the instructions of the surgeon). One month later (4 months postoperatively), he arrived at our emergency department unable to walk and with pain in the revised hip. At clinical presentation, he reported an incident of sudden pain and then falling during normal walking and with no extreme hip movement or rotation. Radiographic control revealed dissociation of the modular stem at the femoral neck–stem interface without dislocation of the head (Fig. 2).

**Fig. 3:**
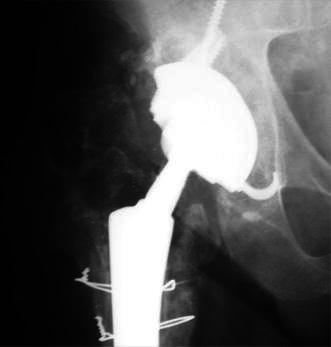
Radiograph after the second operation reveals reimpacted new femoral neck–stem component. Notice the absence of ectopic bone from the lesser trochanter area

Immediate revision surgery was performed to reaffix the neck to the main body of the prosthesis. During the operation, stability test of the acetabular shell revealed adequate fixation of the prosthesis. A new modular interchangeable neck system was implanted; however, as this type of stem also has a modular proximal component, we decided to change it to prevent further complication at the proximal component–stem junction. All intraoperative stability and orientation tests were normal. Postoperative radiographic control was normal (Fig. 3).

The wound healed smoothly after operation, and Harris Hip Score functional score was 90.

## Discussion

**Fig. 4:**
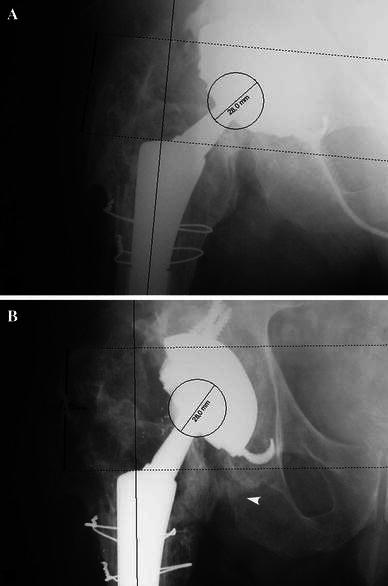
Stem subsidence was measured by processing immediate postoperative (**a**) and 3-month (**b**) follow-up anteroposterior radiographs via Roman v1.7 software (Roman free to share software version V1.70; Robert Jones and Agnes Hunt Orthopaedic Hospital, Oswestry, UK; http://www.Keele.ac.uk/depts./rjah/), as a change in the vertical distance from the proximal tip of the greater trochanter to the shoulder of the stem. Ectopic bone formation at the lesser trochanter area (*white arrowhead*) is noted 3 months postoperatively (**b**)

**Fig. 5:**
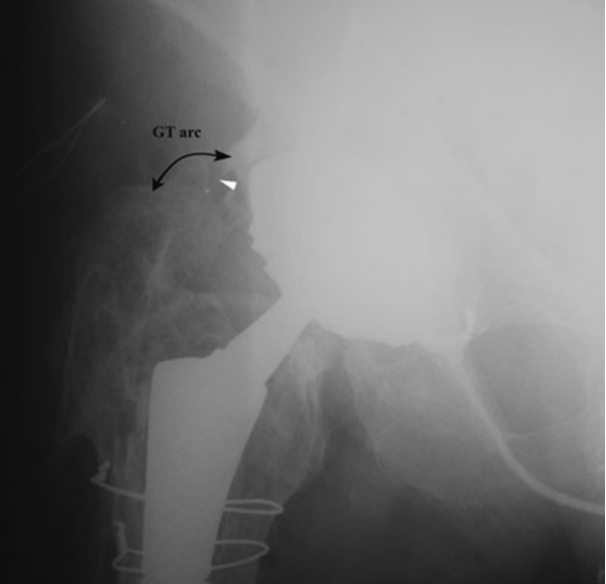
Minimal greater trochanter (GT) arc length and high position of the tip of the GT in relation to the head center predicts early impingement of the GT to the ilium. A bone spur (osteophyte) at the tip of the GT (*white arrowhead*) may further limit impingement-free range of motion

The use of modular components greatly increases flexibility during THA but also introduces the risk of failures at the interfaces and possible intraoperative errors in matching. Dislocation is a potential problem after THA [1, 5, 6], and dissociation of modular components after dislocation is unique to modular systems. Dissociation can occur during closed reduction of dislocation at two different interface levels: the fixed acetabular shell–polyethylene liner interface [2, 7–13], and the femoral head–neck interface [2–4]. In the case reported here, dissociation occurred at the femoral neck–stem interface, with no previous traumatic incidence. To the best of our knowledge, no such case has been reported previously concerning this type of prostheses. The manner in which this incident occurred reveals inadequate modular component fixation or a repetitive force that provoked micromovement of the modular interface that finally led to component dissociation. Potential causes of dissociation during normal walking are:

1. Inadequate orientation of femoral neck resulting in stress forces at the stem–neck interface; in this case, orientation of the femoral and acetabular components cannot be reliably evaluated due to the absence of a computed tomography (CT) scan of the indexed hip.

2. Excessive telescopic movements, which finally lead to dissociation by creating negative pressure in the acetabular area. Computer-assisted measurement of distal stem migration showed a subsidence of 3.6 mm at 3 months, which is considered excessive for this short postoperative period, though it is expected for this type of revision stem and transfemoral approach [14] (Fig. 4). Such an early stem subsidence and subsequent leg shortening can result in loss of intraoperative soft tissue tension and, eventually, in hip-joint instability.

3. Impingement of the femoral neck at the acetabular shell or at osteophytes in the area, causing mechanical stresses at the finally dissociated interface. As mentioned above, component to component impingement cannot be confirmed in our case. However, we consider bony impingement to be more important for this patient. Arc length between the tip of the greater trochanter and the ilium (GT arc) has been shown to correlate with free hip flexion and abduction before impingement [15]. In this case, minimal arc length and the high position of the tip of the greater trochanter in relation to the head center, predicts early bony impingement (greater trochanter to ilium) (Fig. 5). In a computer model, it has been shown that once bony impingement becomes the restricting factor, further changes in implant design and orientation may not improve range of motion (ROM) [15]. Furthermore, in a cadaver study of hip dislocation, osseous impingement was likely to occur between the greater trochanter and the iliac wing before component impingement [16]. Similarly, bony impingement preceded component impingement in about 44% of all conditions tested in a three-dimensional computer model with varying orientations of the femoral and acetabular components [17].

4. Ectopic bone formation causing abnormal movement of the joint. Heterotopic ossification can cause hip-joint instability when the periarticular bone mass limits femoral excursion or contributes to impingement [18]. However, to our knowledge only in two cases was hip dislocation directly attributed to heterotopic ossification [19].

5. Recent retrieval examinations and biomechanical simulation have revealed that modular titanium alloy neck adapters, such as the one used in our case, fail due to surface micromotions [20]. Whether this movement leads, apart from fatigue fracture, to neck dissociation is unclear. Nevertheless, in large case series with similar neck adapters applied, no case of dissociation was reported [21].

In this case, a jumbo cup was used due to extensive bone loss to ensure stable primary fixation. Three cancellous screws were also placed for the same reason. Regarding the femur, the main goal was successful diaphyseal fixation of the stem, so a long, fully porous-coated trapezoid-shaped stem was used. For the modular neck, a straight 0° long neck was selected, allowing fine positioning of the stem in relation to the cup. Although unnecessary [22], three medium hammer blows were applied to fix the neck–stem coupling. Intraoperatively, during the second revision, a large amount of ectopic bone was found in the lesser trochanter area (Fig. 4b), which is a possible cause of stem impingement and, in particular, the neck stem interface, which may lead to dissociation due to repetitive stresses and micromovement in the area. The ectopic bone was removed (Fig. 3), and intraoperative mobilization revealed free movement of the hip joint in all possible directions.

Modular components give the surgeon an intraoperative advantage but also increase the potential for component mismatch and mechanical failure. Dissociation is a rare but possible cause of failure. To prevent this complication, the femoral neck component should be impacted firmly onto the tapered stem base during the operation. Finally, free movement of the joint is essential to prevent abnormal stresses at the interfaces of the modular components.
